# A telephone-based smoking cessation intervention for individuals with COVID-19: A randomized controlled feasibility study

**DOI:** 10.18332/tpc/165826

**Published:** 2023-07-07

**Authors:** Joseph Grech

**Affiliations:** 1 Institute of Applied Sciences, Malta College of Arts, Science & Technology, Paola, Malta; 2 Department for Health Regulation, Health Promotion and Disease Prevention Directorate, Ministry for Health, Pieta, Malta

**Keywords:** COVID-19, smoking cessation, teachable moment, 5As framework, acceptability, cessation induction

## Abstract

**INTRODUCTION:**

Increasing evidence suggests that a diagnosis of a respiratory health condition, such as COVID-19, can prompt a smoker to quit, providing an opportunity to promote and support smoking cessation. However, mandatory quarantine, because of a COVID-19 infection, may stimulate an increase in smoking, making such efforts seem inappropriate or ineffective. This study aimed to investigate the feasibility of a telephone-based smoking cessation intervention for smokers with COVID-19 in Malta.

**METHODS:**

An experimental design with a mixed-methods approach was adopted. Participants (n=80) were recruited from a COVID-19 testing center and equally randomized to the intervention (advised to quit and offered three or four telephone-based smoking cessation support sessions) and control (no intervention) groups. Both groups were asked about their smoking habits at baseline and at follow-up at 1 month and at 3 months. The participants in the intervention group were invited to provide feedback on the intervention using questionnaires and by holding interviews.

**RESULTS:**

Participants were recruited at a rate of 74.1% between March and April 2022. Most participants were female (58.8%), with a mean age of 41.6 years who smoked about 13 cigarettes per day. The majority (75%) accepted the offered smoking cessation support, receiving an average of two to three sessions. Findings indicate that the participants were satisfied with the support, finding it useful for attempting to quit. More participants in the intervention group reported a serious quitting attempt and a 7-day point prevalence abstinence at any point during the first month. However, 7-day point prevalence abstinence rates did not differ at the follow-up at 3 months.

**CONCLUSIONS:**

The study suggests that providing smoking cessation support to individuals with COVID-19 is feasible and well-received. However, the findings suggest that the intervention’s impact may have been brief. Thus, further research is recommended before conducting a conclusive trial.

## INTRODUCTION

The tobacco epidemic remains a significant public health threat in Europe. Tobacco use, such as smoking, which damages nearly every organ system in the human body, increasing the risk of tobacco-associated diseases^1^, kills 0.7 million people each year^2^. While the worldwide age-standardized tobacco smoking prevalence rate amongst those aged ≥15 years is steadily declining, from an estimated 27% in 2000 to 17% in 2020, the World Health Organization (WHO) European Region still reports the highest prevalence rate of tobacco smoking, estimated at 25% in 2020^3^.

Smokers who quit can significantly reduce their risk of developing smoking-attributable diseases and death^4^. While many smokers want to quit smoking, most, however, do not succeed on their own^5,6^. Article 14 of the WHO Framework Convention on Tobacco Control stipulates that WHO member states are to effectively promote and provide comprehensive smoking cessation support^7^. Tobacco users should be identified during health interactions, encouraged to quit smoking in view of their current health or social situation, and referred to or provided with specialized support^5^.

Increasing evidence suggests that a diagnosis of a respiratory health condition can encourage and induce smoking cessation. Pulmonary tuberculosis diagnosis^8^, lung cancer screening and diagnosis^9,10^, and even a diagnosis of an acute or chronic respiratory disease (such as influenza and chronic obstructive pulmonary disease, respectively)^11^, amongst others, have been found to prompt smoking cessation. This is because such health-related events are often seen as ‘teachable moments’, i.e. naturally occurring health events that are thought to prompt individuals to adopt risk-reducing behaviors with the intention to improve their health or reduce their risk of complications^12^.

In the current context, the COVID-19 pandemic, many smokers are apprehensive about the possibility of severe complications if infected^13,14^. Therefore, a diagnosis of COVID-19 may also serve as a ‘teachable moment’, encouraging individuals to cut back or quit smoking to reduce their risk of adverse COVID-19 effects. Smoking impairs the respiratory system’s defence mechanisms by disrupting the epithelial lining, impairing mucociliary clearance, and altering the function of macrophages, neutrophils, and lymphocytes, and hence the ability to contain a COVID-19 infection^15,16^. Conversely, smoking cessation decreases the risk of developing severe adverse outcomes from COVID-19^17^.

Healthcare professionals should therefore make use of the opportunity of such health-related events by promoting and providing smoking cessation support to help such individuals increase their smoking cessation chances and improve their health. However, boredom, stress, and restrictions in movement because of mandatory quarantine (because of a COVID-19 infection) may stimulate an increase in smoking habits^18,19^, possibly making such tobacco cessation efforts seem inappropriate or ineffective.

Given the uncertainty regarding the feasibility of providing smoking cessation support at the time of a COVID-19 diagnosis, a telephone-based smoking cessation intervention was piloted, between March and July 2022, amongst smokers who tested positive for COVID-19 at a testing center facility within the Department for Health Regulation, Health Promotion and Disease Prevention Directorate, Malta. This study aimed to investigate the feasibility of providing this smoking cessation intervention by analyzing recruitment and retention parameters, participants’ feedback on the intervention, and determining the preliminary evidence of the intervention’s effectiveness.

## METHODS

### Design

A two-arm randomized experimental design using a mixed-methods approach was adopted. While a feasibility study may not necessarily be a randomized trial^20^, given that a diagnosis of COVID-19 could prompt the individual to reduce or quit smoking on its own, a comparative analysis of the smoking habits of those who were assigned to the telephone-based smoking cessation intervention and those who were not, helped to determine the preliminary evidence of the intervention’s effectiveness. Quantitative and qualitative acceptability measures were integrated to provide an in-depth understanding of the participants’ views of the intervention for establishing feasibility^21^. The mixing of qualitative and quantitative data followed the convergent design, i.e. quantitative and qualitative data were collected and analyzed separately, and then merged or compared as an additional analysis, followed by an interpretation^22^.

### Participants and setting

The study population consisted of individuals who were aged ≥18 years, had smoked any tobacco products (including novel tobacco products, such as e-cigarettes) within the past seven days, and had been found to be positive for SARS-CoV-2 at the testing center of the Department for Health Regulation, Health Promotion and Disease Prevention Directorate, Malta. Similar to smoking cessation induction trials, which primarily aim to encourage smokers who are not seeking treatment to quit smoking^23^, no participants were excluded from this study based on their lack of intention to quit. Participants were only excluded from the study if they could not be reached by phone.

Guidance from the National Institute for Health Research suggests a sample size of 40 to 50 participants for feasibility trials^24^. However, given that high attrition rates (at 50%) were reported in the systematic review by Matkin et al.^25^ on the use of telephone counseling for smoking cessation, the target sample size was increased to 80. This ensured that the study sample was large enough to give meaningful results but not so large that the trial would take a long time and be costly.

In this testing center, point-of-care Rapid Antigen Tests (RATs) for SARS-CoV-2 were carried out on a daily basis to any self-referred Maltese residents free-of-charge. Following testing, individuals who were found to be positive for SARS-CoV-2 were called and provided with the result on the same day. During the study period, the center coordinators randomly asked patients about their smoking status, and asking identified smokers if they would be interested to participate in the study. On the same or the following day, all interested participants were contacted and further informed about the study by the author, obtaining informed consent. Participants were randomized on a 1:1 ratio to the intervention or the control group using a random sequence list. The random sequence list was computer-generated (in blocks of four) which was then concealed using numbered (sequential) opaque sealed envelopes. The author assigned participants in the order in which they were received, opening each envelope, indicating the participant’s assignment, one at a time.

### Intervention

The telephone-based smoking cessation intervention was based on the 5As and 5Rs framework for smoking cessation^26^, which has been recommended as a standard systematic approach to encourage and support both unmotivated and motivated individuals to quit smoking^5^. After assessing tobacco use, the participants who were assigned to the intervention group were advised to quit smoking in view of the increased risk of COVID-19 complications and asked if they would be interested in being supported to quit smoking. The aim was to encourage the participants to give up smoking as soon as possible, i.e. during their mandatory quarantine period (which lasted 10 days at the time of the study), and to follow them up during and after their quarantine period. As recommended by Matkin et al.^25^ , three (or four) telephone-based smoking cessation support sessions were offered to those willing to attempt to quit smoking. Each session took about 20 minutes, based on the individual’s needs^5^. The algorithm followed is outlined in Supplementary file Figure 1.

The first session took between 20 and 30 minutes. Once participants responded positively to receiving smoking cessation support, their willingness to attempt to quit smoking within the next seven days was assessed, with an emphasis on encouraging them to quit as soon as possible. The 5Rs model was followed for those who were not ready to quit^26^. If after that the participants were still not willing to attempt to quit smoking, no further smoking cessation support was provided. Conversely, the participants who were ready to attempt to quit smoking were supported in developing a quit plan as per the 5As framework^26^. The use of nicotine replacement therapy (NRT) was also suggested. Participants were then provided with a follow-up telephone appointment within the first two days following the quit attempt. During this 20-minute follow-up session, smoking status was re-assessed. Those who did not quit smoking were encouraged to re-attempt quitting as soon as possible and supported to develop another quit plan, and provided with a follow-up appointment within two days following their quit attempt. If they failed again to quit, no further telephone-based support was provided, however, they were invited to seek stop-smoking community services. Conversely, the participants who quit smoking following their first or second attempt, were provided with a 20-minute follow-up session during which they were supported to avoid a relapse, particularly following their quarantine period. They were then provided with a similar but shorter (around 15 minutes) telephone-based follow-up session within a week.

All the telephone-based smoking cessation support sessions were delivered by the author, who is trained in tobacco cessation and has more than ten years of experience in intensive tobacco cessation counseling. To maintain treatment fidelity the author followed a structured guide, based on the 5As and 5Rs framework for smoking cessation^26^.

The participants in the control group were not provided with smoking cessation support. They were, however, asked in detail about their smoking habits and quitting intentions, as for those in the intervention group. At the end of the study period, continuing smokers were encouraged to seek smoking cessation support.

### Measures

Baseline measures included the participants’ sociodemographic and smoking characteristics (Table 1). Feasibility measures focused on demand and implementation, acceptability, and the preliminary assessment of the intervention’s effectiveness^27^.

**Table 1:** Baseline sociodemographic and smoking characteristics of the participants by study condition, recruited from a Maltese COVID-19 testing center in 2022 (N=80)

*Characteristics*	*Randomized (N=80) n (%)*	*Intervention (N=40) n (%)*	*Control (N=40) n (%)*	*p*
**Sex**				
Female	47 (58.8)	20 (50.0)	27 (67.5)	
Male	33 (41.2)	20 (50.0)	13 (32.5)	0.112
**Age** (years), mean (SD)	41.6 (12.90)	42.1 (13.73)	41.1 (12.17)	0.712
**Education level**				
Primary	2 (2.5)	1 (2.5)	1 (2.5)	
Secondary	40 (50.0)	21 (52.5)	19 (47.5)	
Post-secondary	23 (28.7)	12 (30.0)	11 (27.5)	
Tertiary	15 (18.8)	6 (15.0)	9 (22.5)	0.877^c^
**Employment status**				
Employed	57 (71.3)	31 (77.5)	26 (65.0)	
House duties	12 (15.0)	4 (10.0)	8 (20.0)	
Retired	6 (7.5)	3 (7.5)	3 (7.5)	
Unemployed	5 (6.3)	2 (5.0)	3 (7.5)	0.613^c^
**Living alone**				
No	71 (88.8)	36 (90.0)	35 (87.5)	
Yes	9 (11.3)	4 (10.0)	5 (12.5)	1.0^b^
**Living with another smoker**				
No	42 (52.5)	21 (52.5)	21 (52.5)	
Yes	38 (47.5)	19 (47.5)	19 (47.5)	1.0
**COVID-19 signs or symptoms**				
Yes	70 (87.5)	34 (85.0)	36 (90.0)	
No	10 (12.5)	6 (15.0)	4 (10.0)	0.499
**Cigarettes/day**, mean (SD)^a^	12.6 (9.22)	14.6 (9.37)	10.7 (8.77)	0.060
**Minutes before having first cigarette**, median	30.0	30.0	30.0	0.570^d^
**Age at initiation** (years), mean (SD)	16.3 (3.03)	15.7 (2.54)	16.9 (3.39)	0.097
**Quit attempts in past 12 months**				
No	60 (75.0)	30 (75.0)	30 (75.0)	
Yes	20 (25.0)	10 (25.0)	10 (25.0)	1.0
**Importance of quitting** (scale 0–10), mean (SD)^b^	7.9 (2.35)	8.2 (2.14)	7.6 (2.52)	0.236
**Readiness to quit within the next 30 days** (scale 0–10), mean (SD)^b^	5.1 (3.39)	5.5 (3.12)	4.7 (3.63)	0.265
**Confidence to quit within the next 30 days** (scale 0–10), mean (SD)^b^	4.2 (3.24)	4.4 (2.62)	4.0 (3.79)	0.584

a One participant in the intervention group used an e-cigarette, while another participant smoked cigarillos. One participant in the control group used e-cigarettes and smoked cigarettes.

b Motivation rulers^40^. The p values were generated from independent-samples t-test for continuous variables and chi-squared test for categorical variables.

c Fisher's exact test was used for generating the p values as some of the groups were quite small.

d The median test was used as data were not normally distributed.

Demand and implementation measures included the number of identified smokers, the number of smokers who accepted to participate in the study and those offered smoking cessation support, the number of smokers who were lost to follow-up, the number of telephone-based smoking cessation support sessions provided, and the use of additional support strategies including NRT.

Acceptability measures included satisfaction with and perceived usefulness of the provided intervention. These were measured by means of an anonymous online questionnaire and by holding semi-structured telephone interviews, both of which were done at follow-up at 1 month.

All participants who were assigned to the intervention group were asked to fill in the questionnaire which asked about their satisfaction with having been asked about their smoking habits, advised to quit, and offered smoking cessation, using a 5-point Likert scale ranging from ‘very unsatisfied’ to ‘very satisfied’. Those who refused support were asked to state why and those who accepted were asked about their satisfaction with the support provided, using the same Likert scale. Using open-ended questions, they were also asked to state which aspect they were most and least satisfied with. Furthermore, using another 5-point Likert scale ranging from ‘strongly disagree’ to ‘strongly agree’, the participants were asked about their perceived usefulness of the smoking cessation support provided. They were also asked for suggestions for improvement. All participants were asked about their personal characteristics, whether they had seriously attempted to quit (and quit) smoking (defined below), and the number of smoking cessation support sessions provided. The questionnaire was created in English and translated into Maltese by a professional bilingual translator. The Maltese version was back-translated to English by another bilingual translator and compared to the original version, to ensure accuracy. The participants were provided with the link to the English or the Maltese questionnaire, according to their preference.

The qualitative sample was smaller than the quantitative sample, consisting only of a stratified (by sex and by whether they had seriously attempted to quit, and quit smoking) purposeful sample of the participants who were provided with the telephone-based smoking cessation intervention for providing an in-depth understanding of the acceptability of the intervention. To ensure a sufficient sample size, the size was based on the principle of ‘data saturation’, when the new data collected repeated what was already previously expressed, with no reference to new concepts/themes associated with the data^22^. Reference was made to the seminal study by Guest et al.^28^ in which data saturation was relatively achieved after 12 interviews^28^. The sample size was then increased to 15.

The interviews followed a question and probe guide which was based on the questions outlined in the questionnaire. This guide was created in English and translated into Maltese and back-translated, as was done for the questionnaire. All interviews, which lasted around 20 minutes each, were moderated by the author and held in English or Maltese according to the participant’s preference.

To determine the preliminary evidence of the intervention’s effectiveness, participants were asked whether they had seriously attempted to quit smoking (including novel tobacco products) in the first month following their COVID-19 diagnosis (defined as an attempt where the smoker decides to try to never use tobacco again) and whether they reported a seven-day abstinence period following the serious quit attempt (referred to as seven-day floating abstinence)^29^, both of which were assessed at follow-up at 1 month. Furthermore, the self-reported seven-day point prevalence abstinence at follow-up at 1 month and at 3 months, and the biochemically verified abstinence at follow-up at 3 months were also collected^29^. As recommended by Benowitz et al.^30^, biochemical verification of tobacco abstinence was carried out by using a carbon monoxide (CO) monitor (cut-off <6 ppm) and by testing saliva for cotinine exposure using a multilevel lateral flow immunoassay salivary test with a 20 ng/mL cut-off. Continuing smokers were asked about the number of cigarettes smoked per day at follow-up at 1 month and at 3 months.

### Analyses

Quantitative analyses were done using IBM SPSS Statistics Version 27. Descriptive statistics were used to summarize sociodemographic and smoking characteristics and feasibility measures. Intention-to-treat analysis was used; smokers who could not be reached during follow-up were considered non-quitters and non-reducers. Group comparisons for baseline and effectiveness data were done using independent-samples t-test or median test for continuous variables and chi-squared test or Fisher’s exact test for categorical variables, as appropriate.

Qualitative data, from the questionnaire’s open-ended questions and the audio-recorded interviews (which were transcribed verbatim), were analyzed using applied thematic analysis, a rigorous explorative and inductive method for identifying and presenting the meanings of textual data as accurately and comprehensively as possible^31^. Unlike the returned questionnaires, some of the interviews were held in Maltese. These were not translated, to maintain the validity and reliability of the acquired data^31^. Rather, the original text was analyzed and coded, identifying themes/sub-themes in the source language, then translating all identified themes into English^32,33^. All transcripts were imported and analyzed using NVIVO (version 1.7).

To enhance reliability, the bilingual author served as both the primary and secondary coder by reviewing again all themes/codes after some time, to ensure that these reflected the meanings of the textual data^31^. As recommended by Bradshaw et al.^34^, to enhance rigor, in terms of credibility, confirmability, dependability, and transferability, the methods undertaken and data analysis processes were documented and presented (so that this study can be replicated), and the themes and codes identified were solely based on participants’ data and supported by excerpts (English translations of quotes in Maltese are provided).

Following quantitative and qualitative analyses, the results were integrated for validation purposes and for providing a deeper understanding of the acceptability of the intervention. Hence, common concepts from both sets of findings were identified and compared, for confirming, refuting, or expanding each other^22^. This was followed by an interpretation of the integrated findings to answer the study’s objective.

## RESULTS

### Demand and implementation

**Figure 1:**
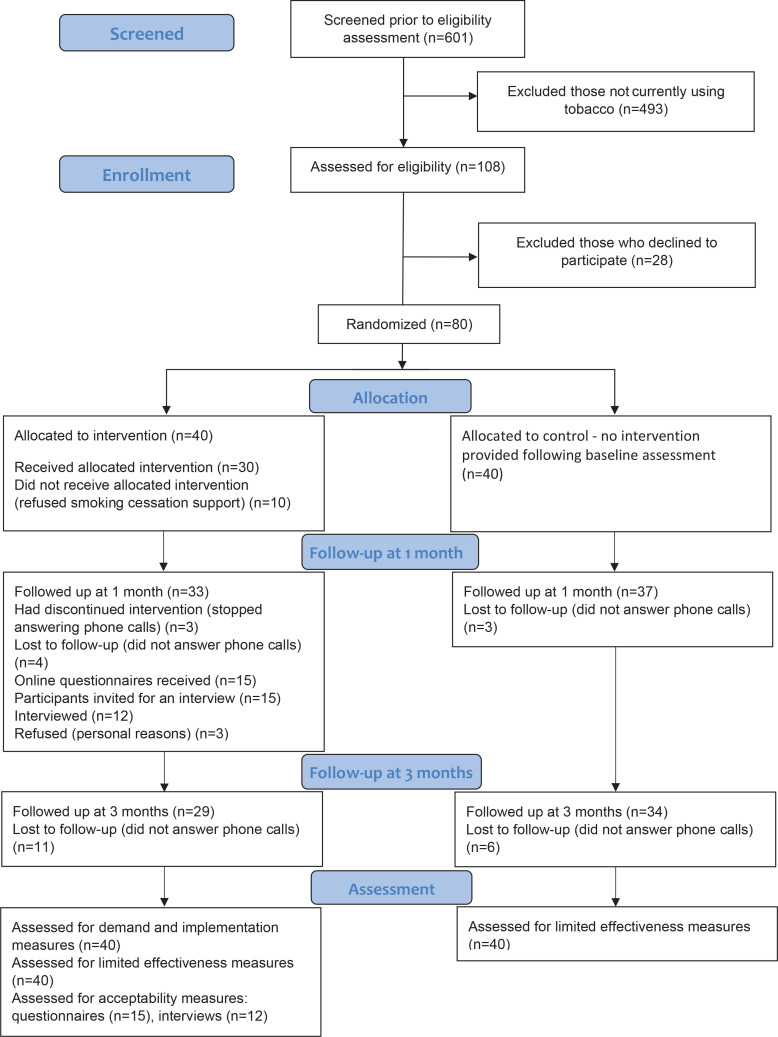
The flow of participant in this feasibility study, recruited from a Maltese COVID-19 testing center in 2022

The selection process of participants in this pilot trial is outlined in Figure 1, based on the CONSORT extension for randomized pilot and feasibility trials flow diagram^35^. Between March and April 2022, 601 individuals with COVID-19 were screened for tobacco use. Out of 108 identified smokers (18.0%), 80 individuals (74.1%) agreed to participate in the study and were equally randomized to the intervention and control groups. Randomization led to similar groups for all baseline characteristics (Table 1). Most participants were female (58.8%), with a mean age of 41.6 years (SD=12.9), who smoked about 13 cigarettes per day. While the participants deemed it important to quit smoking, they were neutral about quitting in the next 30 days, slightly lacking confidence.

On being advised to quit smoking, most participants (n=30; 75%) from the intervention group accepted the offered smoking cessation support. Most participants received two (n=11) or three (n=11) telephone-based smoking cessation support sessions. Five participants were only provided with one session. Three participants, who reported quitting smoking at their second attempt (after their second session), were provided with four sessions each. In addition, NRT was utilized by five participants. At follow-up at 1 month and at 3 months, seven (18.0%) and eleven (28.0%) participants from the intervention group, and three (8.0%) and six (15%) participants from the control group, were lost to follow-up (response rate at follow-up at 3 months was 78.8%). The participants in the control group did not report utilizing NRT or receiving smoking cessation support during the study period. None of the participants in the intervention group sought community-based smoking cessation support during the study period.

### Acceptability (questionnaires)

Fifteen questionnaires were received at follow-up at 1 month (45.5% response rate), three of whom were filled in by participants who had initially refused the offered smoking cessation support. The characteristics of those who responded to the questionnaire are given in Supplementary file Table 1. Table 2 outlines the participants’ responses with regard their satisfaction with the smoking cessation intervention.

**Table 2:** Satisfaction with the smoking cessation intervention, questionnaire responses of the participants from the intervention arm at follow-up at 1 month (N=15)

*How satisfied are you?*	*Rating, n (%)*					*Total n*
	*Very unsatisfied*	*Unsatisfied*	*Neutral*	*Satisfied*	*Very satisfied*	
Having been asked about your smoking habits	0	0	3 (20.0)	4 (26.7)	8 (53.3)	15
Having been advised to quit smoking	0	0	3 (20.0)	4 (26.7)	8 (53.3)	15
Having been offered telephone-based smoking cessation support	0	0	2 (13.3)	3 (20.0)	10 (66.7)	15
With the support you received to stop smoking	0	0	0	4 (33.3)	8 (66.7)	12
The duration of each individual call	0	0	0	2 (16.7)	10 (83.3)	12
The number of calls you had	0	0	0	4 (33.3)	8 (66.7)	12

All respondents who refused the offered smoking cessation support remarked being neutral about having been asked about their smoking habits and advised to quit. Two of these participants were also neutral about having been offered smoking cessation support. However, none of these respondents explained why they refused the offered support. On the other hand, all the other participants were very satisfied or satisfied with the smoking cessation intervention. On being asked about which aspect of the smoking cessation support they were most satisfied with (n=8), four participants remarked on being given support at a critical time, ‘given support when needed the most’ I2 (Female, reported quitting but currently smoking). Three participants remarked on having been attentive to their needs, ‘ready to listen and assist’ I7 (Female, reported quitting but currently smoking), while two participants highlighted the advice given on how to quit smoking, ‘The explanation … on how to quit smoking’ I5 (Female, did not attempt to quit). None of the respondents identified an aspect of the smoking cessation support provided that they were least satisfied with.

As shown in Table 3, most participants strongly agreed or agreed that the smoking cessation intervention was useful. None of the respondents (n=7) provided any suggestions for improvement but rather stated that they were happy with the intervention.

**Table 3:** Perceived usefulness of the smoking cessation intervention, questionnaire responses of the participants from the intervention arm at follow-up at 1 month (N=12)

*The smoking cessation support*	*Rating, n (%)*					*Total n*
	*Strongly disagree*	*Disagree*	*Neutral*	*Agree*	*Strongly agree*	
Was provided at the right time	0	0	0	5 (41.7)	7 (58.3)	12
Met my expectations	0	0	0	6 (50.0)	6 (50.0)	12
Helped me to attempt to quit smoking	0	0	3 (25.0)	4 (33.3)	5 (41.7)	12

### Acceptability (interviews)

Out of the 15 invited participants, 12 consented to be interviewed. The other three refused, stating that they felt uncomfortable being audio-recorded. The characteristics of the interviewees are given in Supplementary file Table 2.

Table 4 outlines the aspects of the intervention which the participants remarked being satisfied with. Most participants (n=9) remarked being satisfied as the support was helpful. Five participants also remarked that it was a pleasant surprise, while four participants stated that the support was attentive.

**Table 4:** Aspects of the intervention which the participants remarked being satisfied with, findings from 12 interviews conducted with participants from the intervention arm at follow-up at 1 month

*Themes and sub-themes*	*Quotes (translated quotes in brackets)*	*Participants’ codes (number of participants)*
**Helpful**		
For smoking cessation support	*‘I think it’s very helpful, and I mean with your help even though we just spoke on the telephone I managed to stop smoking. It helped me a lot.’* (I3; Male, self-reported quitter)	I1, I2, I3, I4, I5, I6, I8, I9, I12 (9)
To get through the quarantine period	*‘During the period of quarantine, I was very, very lonely. Those ten days were like, not hell, like I was in prison basically, and having a person who took care of me helped.’* (I9; Male, self-reported quitter)	I5, I9 (2)
**Pleasant surprise**	*‘I didn’t expect it at all. I didn’t expect this service at all, basically. So really and truly it was welcome.’* (I9; Male, self-reported quitter)	I2, I8, I9, I10, I11 (5)
**Attentive**	*‘… and you were very listening.’* (I8; Female, reported quitting but currently smoking)	I5, I6, I8, I12 (4)
**Encouraging**	*(‘The words you said to me, which kind of encouraged me.’)* (I2; Female, attempted but did not quit)	I1, I2, I7 (3)
**Good follow-up support**	*‘… and then the follow-up was good also. Happy with the follow-up.’* (I11; Female, attempted but did not quit)	I4, I10, I11 (3)

Table 5 displays the aspects of the intervention which were perceived useful by the participants.

**Table 5:** Perceived useful aspects of the intervention, findings from 12 interviews conducted with participants from the intervention arm at follow-up at 1 month

*Themes*	*Quotes (translated quotes in brackets)*	*Participants’ codes (number of participants)*
**Advice on how to quit smoking**	*(‘… by telling me what to do, you helped me quit smoking.’)* (I7; Male, self-reported quitter)	I1, I4, I6, I7, I11, I12 (6)
**Advice on the harms of smoking**	*‘… telling me so, that cigarettes were most likely to affect more the lungs during COVID-19, it hit a nail on the head, basically.’* (I9; Male, self-reported quitter)	I2, I3, I8, I9, I11 (5)
**Being followed up**	*‘I mean even if I was tempted to smoke, I was, I knew that you were going to call, so I held off.’* (I11; Female, attempted but did not quit)	I1, I11 (2)

Most participants remarked finding the advice on how to quit smoking (n=6) and on the harms of smoking (n=5) useful. Two participants remarked that the latter was particularly useful since they were unwell at that time:

*‘The fact that I wasn’t well was a reason, I mean, and then obviously the talk was what I needed to hear.’* I11 (Female, attempted but did not quit)

On its own, feeling unwell was found to encourage abstinence (n=2), however, motivation dropped upon feeling better:

*‘In the first week yes, the second week maybe not … But the first week, yes, especially the way I was feeling … but the second week then I didn’t feel like that. The first week I sort of decided not to smoke anymore.’* I4 (Female, did not attempt to quit; quote translated from Maltese)

Two participants also remarked having found NRT useful.

Nonetheless, several difficulties/challenges to smoking cessation were reported by the participants (shown in Table 3 and Supplementary file Table 1). Most participants highlighted the cravings (n=4), nervousness (n=3), and the quarantine period (n=3). As regards the latter barrier, three participants also expressed mixed feelings about being supported during the quarantine period:

*‘Yes, it could be helpful as well, but knowing how I was, I was very dodgy and quite down … I was just wanting to go out and have it (a cigarette) afterwards.’* I8 (Female, reported quitting but currently smoking)

### Acceptability (integrated analysis)

Both sets of findings confirm one another. Both participants were satisfied with the smoking cessation intervention provided, also perceiving it useful for smoking cessation. In both sets of findings, the participants highlighted the support given, which was found to be provided at a critical time and was attentive to their needs. They also remarked on the advice given to quit smoking. Nonetheless, unlike the participants who accepted the smoking cessation support and filled in the questionnaire, three interviewees expressed mixed feelings about being supported to quit smoking during the quarantine period.

### Preliminary evidence of the intervention’s effectiveness

At follow-up at 1 month, 25 (62.5%) and 11 (27.5%) participants from the intervention and control groups, respectively, reported having seriously attempted to quit smoking (p=0.002). While nine participants (22.5%) in the intervention group versus five participants (12.5%) in the control group reported having been completely abstinent from tobacco for at least seven days following their quit attempt, this was not significant (p=0.239). Five (12.5%) and three (7.5%) participants from the intervention group versus three (7.5%) and four (10%) participants from the control group self-reported abstinence at follow-up at 1 month and at 3 months, respectively. Two of the four participants from the control group (who reported being abstinent from smoking at follow-up at 3 months) reported having specifically stopped smoking because they became pregnant. Of those seven participants who self-reported abstinence at follow-up at 3 months, only three participants underwent biochemical verification, confirming abstinence. There were no significant differences between the mean number of cigarettes smoked per day for continuing smokers at 1 month [intervention: 11.8 (SD=8.89) vs control: 10.9 (SD=9.44); p=0.685] and follow-up at 3 months [intervention: 12.8 (SD=8.23) vs control: 10.9 (SD=9.61); p=0.357].

## DISCUSSION

This feasibility study achieved a modest recruitment rate^36^, successfully recruiting the required sample size in two months. Such a finding was also observed in the study of Taylor et al.^10^ where 79.8% of the identified smokers, who had undergone screening for lung cancer, consented to participate in a randomized trial comparing intensive to minimal intensive telephone-based smoking cessation support. While patient acceptability of proactive smoking cessation support has been reported to vary, from 5.4% to 78%^37^, in this feasibility study, the majority accepted the offered smoking cessation support. Likewise, in the study of Taylor et al.10, most participants (84.2%) from both study arms accepted the interventions presented. Furthermore, similar to Taylor et al.^10^ which reported a 69.9% retention rate at 3 months, this study also obtained a satisfactory response at the follow-up at 3 months.

Many smokers in this study were satisfied with having been provided with opportunistic smoking cessation support, commending certain aspects of the intervention. High satisfaction scores were also reported in Taylor et al.^10^ where 53.0% and 27.3% of the participants reported being very satisfied and somewhat satisfied with the smoking cessation support provided, respectively. Nonetheless, it is worth noting that only 15 participants in this study provided their views via the anonymous questionnaires, warranting caution in the generalization of such findings. It would thus be advisable to conduct further research to establish the acceptability of the study intervention.

This study suggests that a COVID-19 diagnosis is a ‘teachable moment’ for promoting smoking cessation. This study found that when the participants (who were just diagnosed with COVID-19) were provided with smoking cessation advice and support, the majority reported making a serious attempt to quit smoking, and 22.5% of the participants reported being abstinent from smoking for a week. This is in line with the findings of Goel et al.^8^ who found that the provision of a brief smoking cessation intervention at the time of a pulmonary tuberculosis diagnosis and the subsequent follow-up treatment visits was associated with higher abstinence rates when compared to standard care (adjusted Incidence Risk Ratio, IRR: 1.52; 95% Confidence Interval, CI [1.19-1.87]). Health professionals working with COVID-19 patients who smoke should therefore make use of the opportunity of their patients’ current respiratory health situation, encourage them to quit smoking and provide/refer them to specialized smoking cessation support.

Although the sample was not powered to evaluate abstinence rates, the findings obtained suggest that the effect of the intervention may have been short-lived. However, before drawing further conclusions, one should also interpret the results in light of the sample characteristics, the study’s context, and the intervention approach used.

In this study, as in cessation induction trials^23^, the identified smokers were not seeking assistance to quit. Furthermore, they had a neutral stance towards their readiness to quit at baseline. Despite having encouraged most participants to attempt to quit smoking in view of their current condition, it could be that they were no longer motivated to quit/abstain from smoking following recovery or the abatement of signs and symptoms. This was highlighted by two interviewees, who despite reporting feeling motivated to quit smoking on experiencing the COVID-19 signs and symptoms, remarked that their motivation waned on feeling better. To enhance comprehension, future research should investigate the perspectives of smokers who acquire COVID-19 regarding the influence of smoking on COVID-19 prognosis.

The imposed quarantine period may have also limited the participants’ smoking cessation efforts. While most participants reported that the intervention was provided at the right and critical time, remarking that it was a pleasant surprise, some participants identified the quarantine period as having been an additional challenge to quit smoking. Furthermore, mixed feelings about being supported to quit smoking during the quarantine period were also reported. Hence, future research should also investigate the feasibility of providing smoking cessation support to individuals with COVID-19 who may not be on mandatory quarantine.

Unlike in the study of Goel et al.^8^, in this study the intervention had to be delivered remotely. Considering that face-to-face smoking cessation consultations have been proven to be more effective than telephone-based support in assisting smokers to quit during the COVID-19 pandemic (adjusted odds ratio, AOR=1.96; 95% CI: 1.15–3.35)^38^, it would be beneficial for future research to explore the addition of face-to-face smoking cessation support to a telephone-based smoking cessation intervention. In this study, the intervention was also minimal in its intensity. In Taylor et al.^10^ who reported a higher abstinence rate in the intervention group at follow-up at 3 months (OR=2.7; 95% CI: 1.44–5.08), the intervention consisted of eight, and not three to four 20-minute telephone sessions. Furthermore, the participants in the intervention group were provided with an eight-week supply of nicotine patches^10^. Since some participants in this study reported feeling anxious and having cravings when attempting to quit smoking, it is advisable for future research to explore the possibility of supplementing telephone-based smoking cessation interventions with free NRT starter kits. The provision of free NRT starter kits has been associated with increased quit attempts, use of pharmacotherapy for smoking cessation, and higher rates of smoking abstinence compared to standard care, as observed in a recent smoking cessation induction trial^39^.

### Strengths and limitations

The strength of this study is that a mixed-methods approach was adopted to explore the feasibility and acceptability of the study intervention. The findings generated provide a better understanding of the acceptability of the intervention, providing further understanding as to why its effect may have been short-lived. Nonetheless, the interviews were conducted by the author who also delivered the intervention. It is thus possible that the interviewer’s presence may have introduced bias in the responses of the interviewees. To collect more impartial opinions on the acceptability of the study intervention, an online anonymous questionnaire was utilized. Interestingly, the results obtained from both the interview and the questionnaire were found to be consistent with each other, thereby enhancing the credibility and confirmability of the findings^34^. However, the response rate for the questionnaire was low, indicating the need for further research to ascertain the acceptability of the study intervention.

The study’s employment of a randomized experimental design to preliminarily evaluate the effectiveness of the intervention was another positive aspect. Although the self-reported seven-day point prevalence abstinence at the follow-up at 3 months was supported by biochemical verification, the seven-day floating and point prevalence abstinence rates reported at the follow-up at 1 month were not. To improve the validity of the results, future research should also consider confirming the self-reported smoking cessation data through biochemical verification, i.e. at the follow-up at 1 month and possibly following the participants’ serious quit attempt.

## CONCLUSIONS

This study aimed to investigate whether a smoking cessation intervention delivered over the phone could be feasible and also acceptable for individuals with COVID-19. This research showed that the enrolment and retention rates were satisfactory, and that the majority of the participants found the intervention to be beneficial, resulting in most of them making a serious attempt to quit smoking. Additionally, 22.5% of participants reported not smoking for a week. Healthcare professionals working with COVID-19 patients who smoke should therefore seize the opportunity presented by the patients’ current respiratory health situation to encourage them to quit smoking and provide or refer them to specialized smoking cessation support.

While the study’s sample size did not have enough power to assess abstinence rates, the results suggest that the intervention’s impact may have been brief. Thus, some research proposals have been recommended before a conclusive trial can be conducted in the future.

## Data Availability

The data supporting this research are available from the author on reasonable request.
