# Supplementary material for: A telephone-based smoking cessation intervention for individuals with COVID-19: A randomized controlled feasibility study
Source: Tob Prev Cessat. 2023 Jul 7;9:23. doi: 10.18332/tpc/165826 (PMC10326861; doi:10.18332/tpc/165826)
Supplement: Supplementary file 1 [file TPC-9-23-s1.pdf]

Figure 1: Study algorithm for those assigned to the intervention arm (n=40)

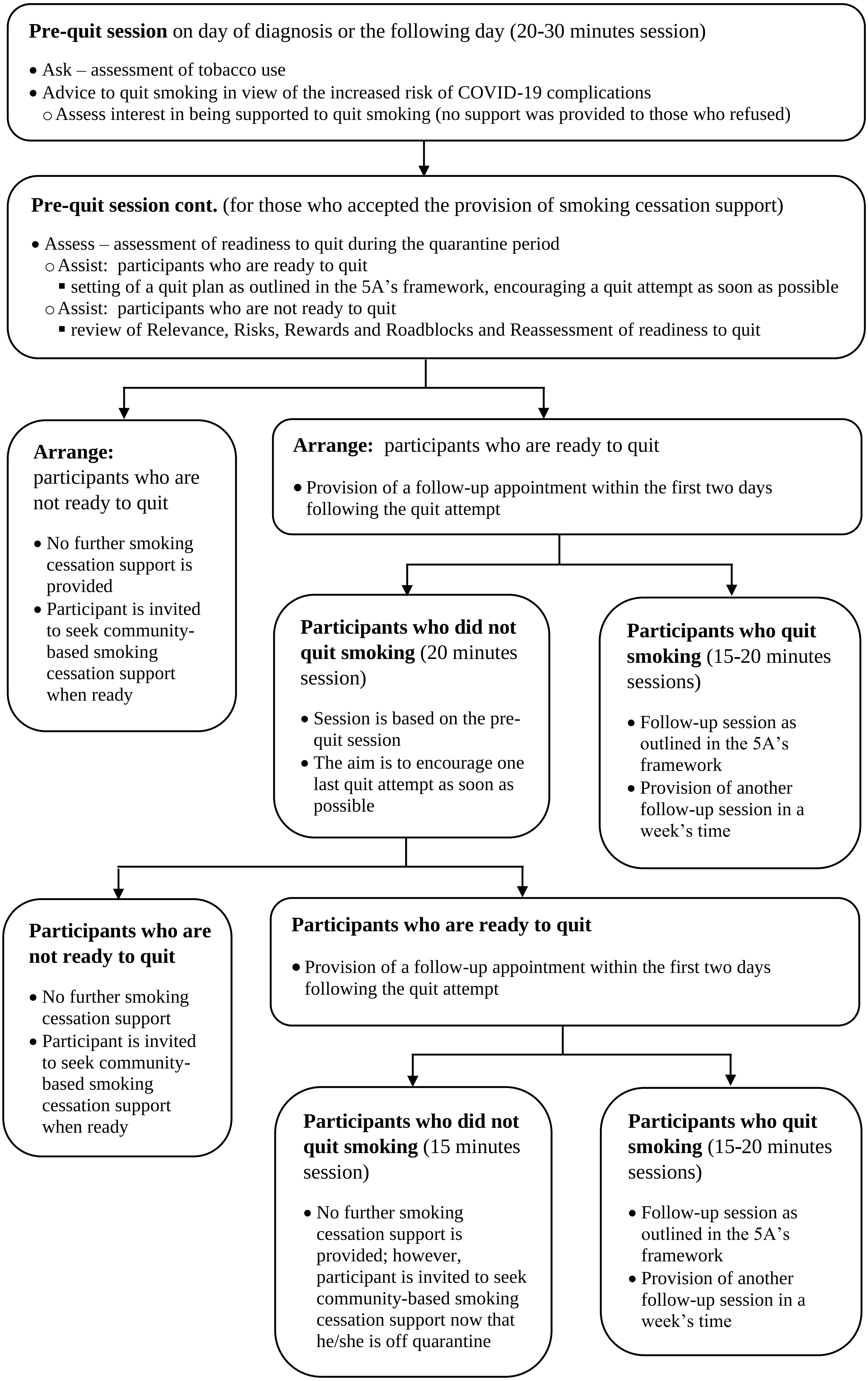

Table 1: Characteristics of the participants (n=15) from the intervention arm who filled in the questionnaire at one-month follow-up

Variable	Response	Value<br>*n*
Sex	Female	10
	Male	5
Median age (in years)		47
Median number of sessions provided		3
Attempted quitting	Yes	10
	No	5
Quit smoking (for at least seven days) following quit attempt	No	11
	Yes	4
Abstinent at one month	No	12
	Yes	3

Table 2: Characteristics of the participants (n=12) from the intervention arm who were interviewed at one-month follow-up

Variable	Response	Value<br>*n*
Sex	Female	8
	Male	4
Median age (in years)		43
Median number of sessions provided		2
Attempted quitting	Yes	10
	No	2
Quit smoking (for at least seven days) following quit attempt	No	9
	Yes	3
Abstinent at one month	No	9
	Yes	3

Table 3: Difficulties and challenges to smoking cessation – findings from 12 interviews (conducted with participants from the intervention arm at one-month follow-up)

**Themes (and sub-themes)**	**Quotes (translated quotes in brackets)**	**Participants' code (no. of participants)**
Cravings	*"Ehh.. the cravings hu for cigarettes, its not as easy as one would think."* I11 (Female – attempted but did not quit)	I7, I10, I11, I12 (4)
Nervousness	(*"the nerves"*) I6 (Female – attempted but did not quit)	I5, I6, I10 (3)
Quarantine period	(*"Hmmm.. it doesn't really help, when you are in quarantine, because you see everything so hard."*) I2 (Female – attempted but did not quit)	I2, I6, I10 (3)
Hectic lifestyle	(*"To quit you can't be like I am now, having so much things to do."*) I4 (Female – did not attempt to quit)	I1, I4 (2)
Smoking routine	*"I have my specific times, where I enjoy smoking and there are specific times where I smoke because I smoke, you know. That was a bit difficult."* I3 (Male – self-reported quitter)	I3, I5 (2)
Stress	(*"Stress, I think."*) I2 (Female – attempted but did not quit)	I2, I4 (2)

© 2023 Grech J.
